# Resting State Networks Related to the Maintenance of Good Cognitive Performance During Healthy Aging

**DOI:** 10.3389/fnhum.2021.753836

**Published:** 2021-11-05

**Authors:** Satoshi Maesawa, Satomi Mizuno, Epifanio Bagarinao, Hirohisa Watanabe, Kazuya Kawabata, Kazuhiro Hara, Reiko Ohdake, Aya Ogura, Daisuke Mori, Daisuke Nakatsubo, Haruo Isoda, Minoru Hoshiyama, Masahisa Katsuno, Ryuta Saito, Norio Ozaki, Gen Sobue

**Affiliations:** ^1^Brain and Mind Research Center, Nagoya University, Nagoya, Japan; ^2^Department of Neurosurgery, Nagoya University Graduate School of Medicine, Nagoya, Japan; ^3^Department of Rehabilitation Medicine, National Hospital Organization, Nagoya Medical Center, Nagoya, Japan; ^4^Department of Neurology, Fujita Health University, Toyoake, Japan; ^5^Department of Neurology, Nagoya University Graduate School of Medicine, Nagoya, Japan; ^6^Department of Psychiatry, Nagoya University Graduate School of Medicine, Nagoya, Japan; ^7^Department of Neurology, Aichi Medical University, Nagakute, Japan

**Keywords:** resting state network, aging, healthy cohort, cognition, delayed recall

## Abstract

**Purpose:** Maintenance of cognitive performance is important for healthy aging. This study aims to elucidate the relationship between brain networks and cognitive function in subjects maintaining relatively good cognitive performance.

**Methods:** A total of 120 subjects, with equal number of participants from each age group between 20 and 70 years, were included in this study. Only participants with Addenbrooke’s Cognitive Examination – Revised (ACE-R) total score greater than 83 were included. Anatomical T1-weighted MR images and resting-state functional MR images (rsfMRIs) were taken from all participants using a 3-tesla MRI scanner. After preprocessing, several factors associated with age including the ACE-R total score, scores of five domains, sub-scores of ACE-R, and brain volumes were tested. Morphometric changes associated with age were analyzed using voxel based morphometry (VBM) and changes in resting state networks (RSNs) were examined using dual regression analysis.

**Results:** Significant negative correlations with age were seen in the total gray matter volume (GMV, r = −0.58), and in the memory, attention, and visuospatial domains. Among the different sub-scores, the score of the delayed recall (DR) showed the highest negative correlation with age (r = −0.55, *p* < 0.001). In VBM analysis, widespread regions demonstrated negative correlation with age, but none with any of the cognitive scores. Quadratic approximations of cognitive scores as functions of age showed relatively delayed decline compared to total GMV loss. In dual regression analysis, some cognitive networks, including the dorsal default mode network, the lateral dorsal attention network, the right / left executive control network, the posterior salience network, and the language network, did not demonstrate negative correlation with age. Some regions in the sensorimotor networks showed positive correlation with the DR, memory, and fluency scores.

**Conclusion:** Some domains of the cognitive test did not correlate with age, and even the highly correlated sub-scores such as the DR score, showed delayed decline compared to the loss of total GMV. Some RSNs, especially involving cognitive control regions, were relatively maintained with age. Furthermore, the scores of memory, fluency, and the DR were correlated with the within-network functional connectivity values of the sensorimotor network, which supported the importance of exercise for maintenance of cognition.

## Introduction

According to Rowe and Kahn, successful aging consists of three principal components: low risk of disease and disease-related disability, maintenance of high mental, cognitive, and physical functions, and continuous engagement with life, which includes relations with others and productive activity (Rowe and Kahn, 1987, 1997, 2015). During the last two decades, worldwide life expectancy has increased by more than 6.6 years, while healthy life expectancy (HALE), an average period of life-time spent without limitation in daily activities, has also increased by 5.4 years (World Health Organization, 2020). Especially in Japan where the highest aging rate was recorded in the world, the increase in the HALE has exceeded the one in life expectancy (Cabinet Office Japan, 2020). Not only mortality has kept declining, but also years lived with disability has been drastically reduced. Under this global situation, successful aging has gained its importance, and has greatly affected a variety of fields including health science, sociology, economics, and politics.

Cognitive function is an extremely important factor influencing successful aging in the elderly people. It is widely known that cognitive function gradually declines over age even in people who seemed to be healthy. This is especially the case for memory and fluid intelligence, acquired in order to adapt to various circumstances including speed processing, reasoning, working memory, and short term memory (Park et al., 2002). On the other hand, crystalized intelligence, acquired from one’s accumulated experience and education and included language abilities, comprehension, and insight, is maintained or improved with age (Baltes et al., 1999). Empirically, when a screening test for cognitive function is performed, unexpected variations in sub-scores can be observed to some extent even if subjects are considered normal in cognitive function based on the total score falling within the normal range.

Morphological studies of the brain using structural magnetic resonance imaging (MRI) have reported wide range of gray matter volume (GMV) decreases with age (Good et al., 2001; Giorgio et al., 2010). The GMV begins to decrease in early adulthood, and continues to decrease approximately linearly throughout the lifespan (Ge et al., 2002; Sowell et al., 2003; Lehmbeck et al., 2006). Although the GMV is generally reduced with age during healthy aging, it still remains unclear whether a cognitive function decline parallels GMV decline. Several studies have been performed about the associations between regional GMV and cognitive scores, but there is no detailed report on the comparison between subtle changes of cognitive test scores in healthy aging and the changes in GMV.

In network analysis using resting state functional MRI (rsfMRI), reduction of the functional connectivity within the default mode network (DMN) with age has been reported in many literatures (Damoiseaux et al., 2008; Koch et al., 2010; Jones et al., 2011). In addition, a within-network decline in functional connectivity has also been reported in other large-scale functional networks, including the salience network (SN), executive control network (ECN), attention network, sensori-motor network (SMN) and the visual network (VN) involved in primary processing (Onoda et al., 2012; Tomasi and Volkow, 2012; Betzel et al., 2014; Geerligs et al., 2015; Huang et al., 2015). Although such canonical networks showed decreases of within-network connectivity, between-network connectivity of some pairs of these networks somewhat increases (Meier et al., 2012; Betzel et al., 2014; Chan et al., 2014; Bagarinao et al., 2019), a possible reflection of the functional network reorganization with aging. Several studies have also reported the relationship between cognitive decline and network changes, e.g., between anterior DMN and executive control function (Damoiseaux et al., 2008), between SN and configuration ability and frontal lobe function (Onoda et al., 2012), and between cingulate network and episodic memory, attentional function, and executive function (Hausman et al., 2020). However, the target age and the number of subjects included were limited in each study, and the findings were inconclusive.

What are the different factors influencing successful aging? Can these factors be identified based on the characteristics of brain-imaging-derived metrics such as brain volume and connectivity? The purpose of this study was to identify such characteristics by investigating the relationship among aging, brain volume, brain network changes, and cognitive function in healthy subjects. For this purpose, healthy individuals who maintained relatively good cognition were enrolled in the study. Within age groups, ranging from 20 to 70 years, an equal number of subjects were included. Although voxel based morphometry (VBM) analysis was performed as the first step, network analysis using rsfMRI represented the main part of this study. RsfMRI is a useful method to visualize various large-scale networks in the brain by examining the synchronization of the blood oxygen level dependent (BOLD) changes in different brain regions during rest, i.e., without performing any tasks, and has been utilized in evaluating changes in brain networks in aging (Bagarinao et al., 2020) and the pathology of various diseases in the central nervous system from Alzheimer’s disease to brain tumors (Greicius et al., 2004; Maesawa et al., 2015; Mulders et al., 2015; Putcha et al., 2015; Abbott et al., 2016). Our hypotheses are as follows: (1) Even in healthy subjects with total score of the cognitive screening test within normal range, some variations of the sub-items in the cognitive test may reflect association with aging. (2) Such sub-items may have a spatiotemporal relationship with the brain’s structural differences with age. Some sub-items may show differences with age that parallel with the structural differences, whereas others may show the maintenance of these scores, independent from the structural differences. (3) To help us understand such maintenance, a network-based approach may be necessary aside from the morphological approach, and the analysis using rsfMRI may contribute to the evaluation of the alterations of the RSNs, which may be correlated with the subtle change of cognition observed in our healthy cohort. (4) On the other hand, some networks may be maintained despite of advancing age, which may provide an explanation for the neuronal basis of the maintenance of cognitive function. Through these analyses, we will identify the different conditions necessary for the maintenance of good cognitive function during aging, that is, the different conditions for successful aging.

## Materials and Methods

### Participants

This study was part of the on-going healthy aging cohort study in the Brain & Mind Research Center (BMRC) in Nagoya University, which was approved by the Ethics Committee of Nagoya University Graduate School of Medicine (approval number 2014-0068), and conducted following the Ethical Guidelines for Medical and Health Research Involving Human Subjects as endorsed by the Japanese Government. All participants were healthy volunteers who joined in response to the recruitment using leaflets and the website of the BMRC. Inclusion criteria for the original project were as follows: older than 20 years, not pregnant, had no episode for MRI contraindications, no brain diseases such as cerebrovascular diseases, brain tumor, head injury, depression, and schizophrenia. They provided written informed consent before joining the study. Between 2014 and 2020, more than 1,000 volunteers participated. From the pool of volunteers, a total of 120 participants, consisting of 10 men and 10 women in each of the 6 age groups, 20s, 30s, 40s, 50s, 60s, and 70s, were randomly chosen. Exclusion criteria were as follows: (1) inability to complete the Japanese version of Addenbrooke’s Cognitive Examination-Revised (ACE-R) assessment, (2) presence of structural abnormalities (e.g., asymptomatic cerebral infarction, benign brain tumor, white matter abnormalities, etc.) in structural MRI as identified by Japanese board-certified neurologists (HW, KH, and KK) and neurosurgeon (SM), (3) ACE-R total score less than 83, and (4) incomplete imaging data. The mean age for all participants was 48.9 ± 17.6 (SD) years, 22.7 ± 2.0 years old for those in the 20s (*n* = 20), 34.7 ± 2.9 years old in the 30s (*n* = 20), 44.5 ± 2.7 years old in the 40s (*n* = 20), 53 ± 2.7 years old in the 50s (*n* = 20), 63.9 ± 2.7 years old in the 60s (*n* = 20), and 74.6 ± 3 years old in the 70s (*n* = 20). The average number of years for education was 14.24 ± 2.52 years. The percentage of participants who smoked were 58.3% in men, 25% in women, and 41.7% in total (Table 1). In term of head motion, which typically affect the estimation of the functional connectivity, the mean frame-wise displacement (FD) values (Power et al., 2012) was 0.18 + 0.069 mm on average. The number of subjects with mean FD greater than 0.2 mm was 39 (32.5%) and those with less than 0.2 mm was 81 (67.5%). No participants had mean FD greater than 0.5 mm.

**TABLE 1:** The characteristics of participants and the score of ACE-R test.

	All (*n* = 120)		Male (*n* = 60)		Female (*n* = 60)		20s (*n* = 20)		30s (*n* = 20)		40s (*n* = 20)		50s (*n* = 20)		60s (*n* = 20)		70s (*n* = 20)	
	mean	SD	mean	SD	mean	SD	mean	SD	mean	SD	mean	SD	mean	SD	mean	SD	Mean	SD
Age	48.9	17.6	48.6	17.6	49.1	17.8	22.7	2	34.7	2.9	44.5	2.7	53	2.7	63.9	2.7	74.6	3
The year of education	14.2	2.5	14.5	2.7	14	2.4	15.9	1.4	15	2.8	14.4	3.3	12.9	1.9	14.7	1.8	12.7	2.2
ACE-R total (100)	95.7	3.2	95.9	2.9	95.6	3.6	96.6	1.7	97.4	2.6	96.3	2.4	96.5	2.8	95.2	3.5	92.6	3.8
Attention/orientation (18)	17.9	0.4	17.9	0.3	17.9	0.4	18	0.2	18	0	18	0.2	17.9	0.4	18	0	17.5	0.7
Fluency (14)	13.4	1.2	13.4	1	13.5	1.3	13.8	0.4	13.4	1.4	13.4	1	13.4	1.6	13.3	1.2	13.5	1
Memory (26)	23.6	2	23.7	2.1	23.6	2	24.1	0.9	24.9	1.3	24.2	1.2	24.1	1.6	23.2	2.1	21.5	2.8
Language (26)	25.1	1.1	25.3	0.9	25	1.2	25	1.4	25.4	1	25.3	1	25.4	0.7	25.2	0.8	24.7	1.3
Visuospatial (16)	15.7	0.7	15.7	0.7	15.7	0.7	15.8	0.6	15.9	0.5	15.6	0.7	15.8	0.4	15.5	0.8	15.5	0.9
The rate of smoker	41.7%		58.3%		25%		15%		35%		65%		40%		55%		40%	

### Acquisition of MR Imaging Data

T1 anatomical images and rsfMRI data were obtained from all participants. MRI scanning was performed using a Siemens Magnetom Verio (Siemens, Erlangen, Germany) 3.0-T scanner with a 32-channel head coil at the BMRC in Nagoya University. The high-resolution T1-weighted images (T1-WI) were acquired using a 3D magnetization prepared rapid acquisition gradient echo (MPRAGE) sequence with the following imaging parameters: repetition time (TR) = 2.5 s, echo time (TE) = 2.48 ms, 192 sagittal slices with a distance factor of 50% and 1-mm thickness, field of view (FOV) = 256 mm, 256 × 256 matrix size, and an in-plane voxel resolution of 1 × 1 mm2. For the rsfMRI data, a gradient-echo (GE) echo-planar imaging (EPI) sequence was used with the following acquisition parameters: TR = 2.5 s, TE = 30 ms, 39 transversal slices with a 0.5-mm inter-slice interval and 3-mm thickness, FOV = 192 mm, 64 × 64 matrix dimension, flip angle of 80° and 198 total volumes. During rsfMRI scan, the participants were instructed to close their eyes but to stay awake. The subject’s head was tightly fixed with cushions to minimize its motion.

### Neuropsychological Test

A Japanese version of ACE-R was performed to evaluate cognitive function for all participants. ACE-R is a brief battery that provides evaluation of five cognitive domains (orientation / attention, memory, verbal fluency, language and visuospatial ability) with a total score of 100 points, and usually requires about 15 min for the examination (Mathuranath et al., 2000; Yoshida et al., 2012). Participants who obtained 82 points or less in total score were excluded from this study because of the possibility of dementia. The sensitivity and specificity of the total ACE-R score was reported to be 99 and 99%, respectively, for dementia when the cut-off score of 82/83 was used, and 87 and 92%, respectively, for MCI, when the cut-off score of 88/89 was used (Yoshida et al., 2012). In addition to the total score, the scores for each of the five cognitive domains, the sub-score of verbal fluency such as semantic or phonological word recall, the sub-score of memory such as memorization, delayed memory, and recognition, and the sub-scores for others were also documented.

### Image Preprocessing

Image preprocessing for the anatomical T1WI and rsfMRI dataset was performed using Statistical Parametric Mapping (SPM12, Wellcome Trust Center for Neuroimaging, London, United Kingdom) running on Matlab (R2016a, MathWorks, Natick, Mass, United States). The T1WI images were first segmented into component images including gray matter (GM), white matter (WM), and cerebrospinal fluid (CSF), among others, by the segmentation approach included in SPM12. Bias-corrected T1WI and the transformation information from subject space to MNI (Montreal Imaging Institute) space were also obtained during segmentation. For rsfMRI dataset, we excluded the first 5 volumes in the series in order to account for the effects of the initial scanner inhomogeneity. Slice-time correction was then performed relative to the middle slice (slice 20), and the images were realigned to the mean functional volume. The mean volume, together with the realigned functional images, were then co-registered to the bias-corrected T1WI anatomical images. The co-registered functional images were normalized to the MNI space using the transformation information obtained during segmentation, resampled to have an isotropic voxel resolution equal to 2 x 2 x 2 mm^3^, and smoothed using an isotropic 8-mm full-width-at-half-maximum (FWHM) 3D Gaussian filter. To correct for head motion and contribution from other nuisance signals, we regressed out 24 motion-related regressors [R_t_ R_t_^2^ R_t–1_ R_t–1_^2^], where R = [x, y, z, roll, pitch, yaw] represents the estimated motion parameters (3 translations and 3 rotations). Signals extracted from spherical ROIs within the CSF (center’s MNI coordinate = [20, −32, 18], radius = 4 mm) and WM (center’s MNI coordinate = [24, −12, 34], radius = 4 mm), the global signal, as well as the signals’ derivatives were also removed. Finally, the preprocessed data were then bandpass filtered within 0.01–0.1 Hz. All preprocessing were performed using in-house Matlab scripts as reported previously (Bagarinao et al., 2019). The preprocessed dataset were used in the succeeding analysis.

### Data Analysis

#### A Correlation Analysis and Regression Analysis for Age-Related Factors

In order to identify the factors related to aging, correlation analyses with age were performed using Spearman’s rank correlation coefficient method. Variables individually examined included gender, years of education, GMV, WMV, CSFV, and total intracranial volume (TICV) calculated from the anatomical T1WI images, the total score of ACE-R, and the sub-score of each domains (orientation / attention, memory, verbal fluency, language and visuospatial ability). In addition, sub-items of cognitive function in the ACE-R were also examined. Considering the ceiling effects, only sub-items with relatively high variance (SD > 0.5), such as the counts of the correct answer to the serial subtraction of number 7, phonological or semantic word recall score, picture naming score, and delayed recall (DR) score, were included in the analysis. The threshold for statistical significance was set at *p* < 0.05. Next, regression analysis was performed for each factor with significant correlation with age. The statistical significance threshold was set at *p* < 0.05. We examined two regression models. One is linear in age, and the other is quadratic. The appropriate regression model (linear vs. quadratic) was assessed using the coefficient of determination (R2), the Bayesian information criterion (BIC), and the Akaike’s information criterion (AIC).

### VBM Analysis With Factors Associated for Aging

The total volumes of GM, WM, and CSF were calculated using the segmented components of the T1-weighted images. Using SPM 12, multiple regression analysis was performed with covariates including age, gender, years of education, and five cognitive domains (orientation/attention, fluency, memory, language, and visuospatial). Global calculation was performed using TICV. The threshold for statistical significance was set at a corrected *p* < 0.05 with a family wise correction (FWE). We also examined the association between GMV and the score of the DR, which showed the highest significant relationship with age in the above correlation analysis, under two different conditions. In one condition, age and the TICV were included as covariates, while in the other condition, age was excluded. Xjview^1^ was used to examine regions with significant association with age or the score of delayed memory in the resulting statistical maps. Automatic anatomical labeling (AAL) was used for the anatomical name of the identified region.

### Resting State Network Analysis for Aging and the Associated Factors

To evaluate the relationship between factors associated with aging and brain functional networks, we used dual regression analysis. The preprocessed rsfMRI datasets from the 120 subjects were temporally concatenated, and group independent component analysis (ICA) was performed using the MELODIC software from the FSL package (Jenkinson et al., 2012). Thirty independent components (ICs) were derived across the whole sample, extracted, and visually compared to a set of reference RSN templates^2^ (Shirer et al., 2012) to identify several well-known RSNs. In dual regression analysis (Filippini et al., 2009), the extracted group ICs were used as spatial regressors and the temporal dynamics associated with each IC for each subject were estimated. These time courses were then used as temporal regressors in a second regression analysis to generate subject-specific maps associated with each group IC. Using the constructed subject-specific maps, regression analysis was performed with the cognitive function scores, year of education, age, gender, and GMV set as regressors. For the cognitive functional scores, in one condition, the scores of the five domains (attention/orientation, fluency, memory, language, visuospatial) were used. In another condition, the DR score was used instead of the memory score. Statistical analysis of each component map was performed using a non-parametric permutation test (5000 permutations), and regions with connectivity showing statistically significant association with each respective factors were identified. All statistical maps were corrected for multiple comparisons using FWE correction with threshold free cluster enhancement. Statistically significance was set at *p* < 0.05.

## Results

### A Correlation Analysis and Regression Analysis for Age-Related Factors

In the correlation analysis using a Spearman’s rank correlation coefficient, the CSFV was the only factor which showed a significant positive correlation (r = 0.55). On the other hand, significant negative correlations with age were seen in the GMV (r = −0.58), DR score (r = −0.55), ACE-R total score (r = −0.36), attention / orientation score (r = −0.35), memory score (r = −0.38), and visuospatial ability score (r = −0.18) (Table 2). The other domains, language and fluency, were not significantly correlated with age. The education year also demonstrated negative correlation with age, which reflect the relatively high college enrollment rate in younger generation and was, therefore, excluded for further regression analysis.

**TABLE 2:** A summary of results in correlation analysis.

	Basic information		ACE-R Scores (total and 5 domains)						Subscores of ACE-R					Morphologic information			
				
	Age	Education year	Total score	Attention/orientation	Fluency	Memory	Language	Visuospatial	Serial 7	The score of verbal fluency: phonological	The score of verbal fluency: semantic	Naming	Delayed recall	GMV	WMV	CSFV	TICV
Mean	48.9	14.2	95.7	17.9	13.4	23.6	25.1	15.7	4.6	6.7	6.7	11.6	6.2	637.5	432.5	346.4	1416.3
Min.	20	9	83	16	7	15	22	13	1	2	3	8	2	409	298.8	155.6	1058.1
Max.	78	21	100	18	14	26	26	16	5	7	7	12	7	806.6	562.1	803.4	1679.1
S.D.	17.63	2.52	3.24	3.87	1.17	2.02	1.05	0.67	0.75	0.8	0.7	0.8	1.16	75.37	49.54	94.54	140.04
**Correlation with age**																	
Correlation coefficient; r	N/A	−0.36	−0.36	−0.35	−0.07	−0.38	−0.14	−0.18	−0.15	−0.02	−0.11	0.06	−0.55	−0.58	−0.13	0.55	−0.23
Significant level		<0.01**	<0.01**	<0.01**	0.439	<0.01**	0.132	0.045*	0.092	0.801	0.222	0.548	<0.01**	<0.01**	0.175	<0.01**	0.802

**FIGURE 1:**
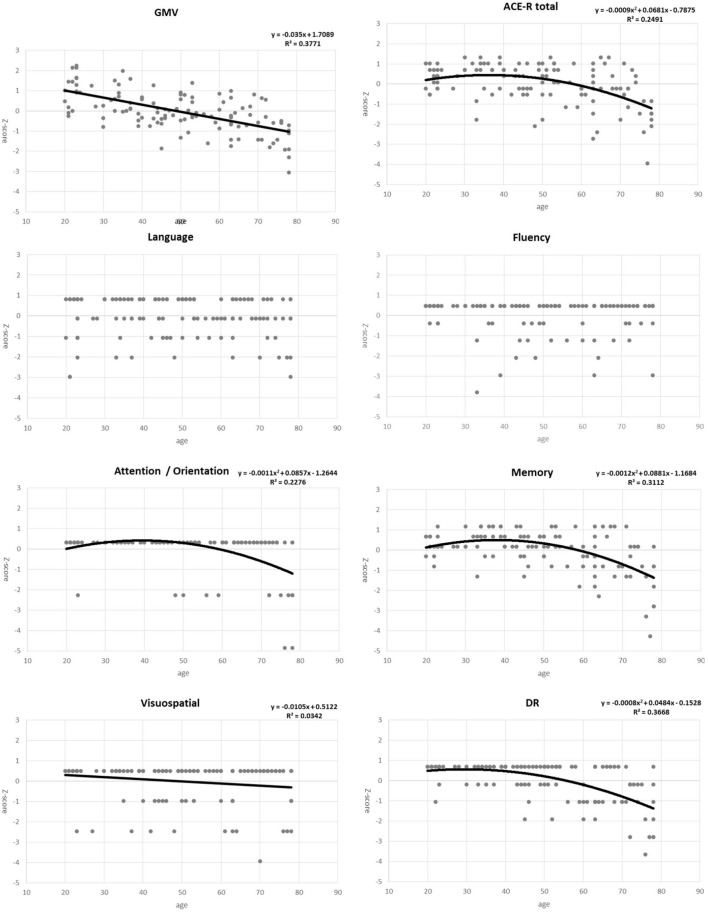
Plots for gray matter volume (GMV), total ACE-R score, five domains of the ACE-R (attention/orientation, memory, fluency, visuospatial, and language), and the delayed recall (DR) score as functions of age. Points represent actual data, whereas solid line/curves represent regression functions.

**FIGURE 2:**
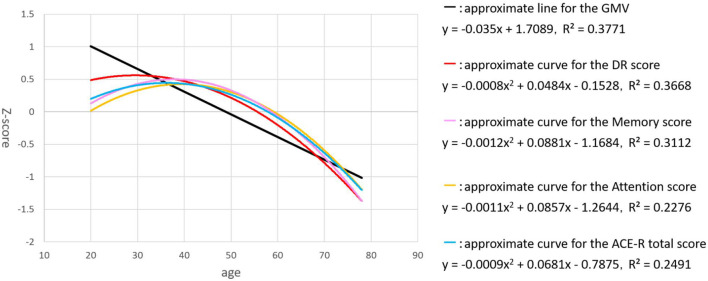
The plots for GMV (black), the DR score (red), ACE-R total score (blue), attention / orientation (yellow), and memory (pink), as functions of age are shown. The vertical axis showed the volume and the score of each subject as a standardized z-score, and the horizontal axis is age.

For the regression analysis of each factor with age, we examined two regression models - linear and quadratic. Individual plots for the different factors examined are shown in Figure 1, whereas the combined plots for GMV, the DR score, ACE-R total score, attention / orientation, and memory, as functions of age are shown in Figure 2. The vertical axis showed the volume and the score of each subject as a standardized z-score, and the horizontal axis is age. The appropriate regression model, shown as solid line, was identified using both AIC, BIC, and R2. The best model was linear for GMV and visuospatial and quadratic for the DR, memory, attention / orientation, and ACE-R total. AIC, BIC, and R2 values for the two regression models of each factor are summarized in Table 3. Among the different cognitive function scores, the DR score showed the highest significant relationship with age. GMV showed a relatively steeper slope from the 20s, and fell below the average value (z - score = 0) at around the age of 50. On the other hand, the DR, total ACE-R score, memory and attention score are relatively stable until late 50’s and decline sharply afterward. The score of visuospatial ability showed mild linear change with age, but the R2 value was small, and its change was not reliable. The other two cognitive domains (language and fluency) did not show significant relationship with age.

**TABLE 3:** A summary of AIC, BIC, and R2 values for the two regression models of each factor.

	AIC		BIC		R2	
Factor	Linear	Quadratic	Linear	Quadratic	Linear	Quadratic
Attention	327.94	315.55	333.52	323.91	0.1292	0.2276
Fluency	343.94	344.03	349.52	352.39	0.0050	0.0207
Memory	317.93	301.81	323.51	310.17	0.1989	0.3112
Language	343.43	340.02	349.00	348.38	0.0093	0.0529
Visuospatial	340.37	342.37	345.94	350.73	0.0342	0.0342
ACERTotal	321.41	312.16	326.98	320.52	0.1754	0.2491
GMV	287.73	289.72	293.31	298.08	0.3771	0.3772
DRscore	299.88	291.70	305.46	300.07	0.3108	0.3668

### VBM Analysis With Factors Associated With Aging

**FIGURE 3:**
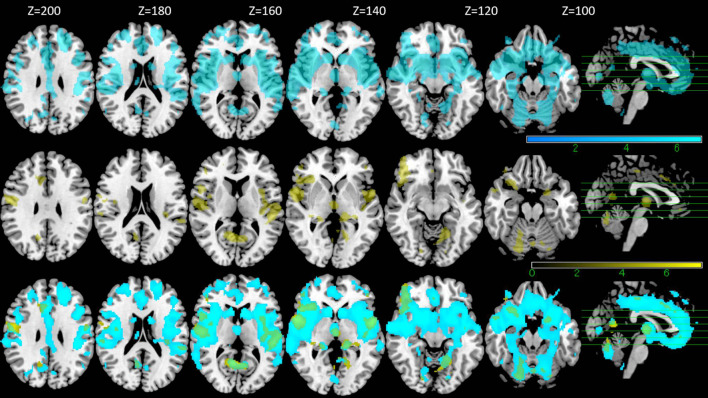
VBM results. Regions with negative correlation with the age are shown in the upper row (blue), those with positive correlation with DR score without age as a covariate are shown in the middle row (yellow) and the overlapped regions between the two are shown in the bottom row (green) (FWE *p* < 0.05).

With VBM, a strong negative correlation with age was observed in many regions across the cerebral cortex. The maximum negative correlation was found in the right posterior central gyrus. Areas with negative correlation with age were widespread and bilaterally observed in the lateral frontal cortices, the lateral temporal cortices, the lateral occipital cortices, the parietal cortices, the cingulate gyrus, the areas surrounding the intraparietal sulcus, and the medial temporal areas including the hippocampus (Table 4, upper row in Figure 3). In VBM analysis for 5 cognitive domains (attention/orientation, fluency, memory, language, visuospatial ability) and education with age and TICV as covariates, no region survived statistical significance.

**TABLE 4:** Anatomical structures correlated with age in VBM.

Peak MNI coordinate	Peak anatomical structure	Peak *T*-value
‘34 −26 48	Postcentral_R	10.49
**Anatomical structures of the clusters (voxel count>100)**		
Frontal lobe	Postcentral_L (1781), Postcentral_R (1350), 1278, Frontal_Mid_L (1278), Frontal_Sup_L (1039), Frontal_Inf_Orb_R (1038), Rolandic_Oper_R (983), Frontal_Mid_R (983), Precentral_R (900), Frontal_Inf_Orb_L (807), Frontal_Sup_R (803), Frontal_Inf_Tri_L (796), Rolandic_Oper_L (785), Precentral_L (730), Frontal_Inf_Oper_R (679), Frontal_Inf_Oper_L (676), Frontal_Inf_Tri_R (558), Rectus_R (506), Rectus_L (451), Frontal_Med_Orb_R (323), Frontal_Mid_Orb_R (253), Olfactory_R (228), Frontal_Sup_Orb_L (212), Frontal_Sup_Medial_L (158), Frontal_Med_Orb_L (154), Frontal_Sup_Orb_R (148), Supp_Motor_Area_R (146), Olfactory_L (127), Frontal_Sup_Medial_R (120)	
Temporal lobe	Temporal_Sup_R (2051), Temporal_Sup_L (1876), Temporal_Mid_R (1265), Temporal_Mid_L (873), Temporal_Pole_Sup_L (693), Temporal_Pole_Sup_R (661), Fusiform_R (636), Fusiform_L (571), Temporal_Pole_Mid_R (255), Heschl_R (242), Temporal_Inf_R (225), Heschl_L (210)	
Parietal lobe	Lingual_L (266), Lingual_R (245), Calcarine_L (199), Calcarine_R (141)	
Occipital lobe	Lingual_L(1332), Lingual_R(1317), Calcarine_L(1002), Calcarine_R(701), Cuneus_L(342), Cuneus_R(312)	
Limbic / Insula	Insula_L (1776), Insula_R (1690), Cingulum_Mid_R (1482), Cingulum_Mid_L (1217), Cingulum_Ant_L (770), Cingulum_Ant_R (689), Hippocampus_L (572), ParaHippocampal_R (348), Hippocampus_R (272), ParaHippocampal_L (238), Amygdala_R (222), Amygdala_L (173)	
Subcortical structures	Putamen_R (766), Putamen_L (655), Caudate_L (575), Caudate_R (536), Thalamus_L (195), Thalamus_R (113)	
Cerebellum	Cerebelum_6_R (1060), Cerebelum_6_L (1012), Cerebelum_Crus1_L (413), Cerebelum_8_L (408), Cerebelum_Crus1_R (396), Cerebelum_4_5_L (357), Cerebelum_Crus2_R (352), Cerebelum_4_5_R (295), Cerebelum_Crus2_L (278), Cerebelum_9_L (213), Cerebelum_8_R (197), Vermis_8 (185), Cerebelum_7b_L (150), Vermis_7 (131)	

VBM analysis for DR and with TICV as the covariate showed positive correlation between DR and gray matter in a relatively large area including bilateral frontal cortices, bilateral temporal cortices, bilateral insular cortices, and bilateral cingulate cortices (*p* < 0.05, FWE) (Table 5, middle row in Figure 3). These regions overlapped with the part of the areas showing negative correlation with age (lower row in Figure 3). However, in the analysis where the age was also included as a covariate, no region survived.

**TABLE 5:** Anatomical structures correlated with age in VBM.

Peak MNI coordinate	Peak anatomical structure	Peak *T*-value
‘−56 2 2	Temporal_Sup_L	7.48
**Anatomical structures of the clusters (voxel count>100)**		
Frontal lobe	Frontal_Inf_Orb_R (664), Precentral_R (348), Rolandic_Oper_R (346), Rolandic_Oper_L (309), Postcentral_R (269), Frontal_Inf_Oper_R (159), Frontal_Inf_Tri_R (154), Frontal_Mid_Orb_R (144)	
Temporal lobe	Temporal_Sup_L (588), Temporal_Sup_R (225), Heschl_L (145), Temporal_Pole_Sup_R (111), Heschl_R (105)	
Parietal lobe	Precuneus_R (142)	
Occipital lobe	Lingual_L (322), Calcarine_R (181), Calcarine_L (179), Lingual_R (127)	
Limbic / Insula	Insula_R (525), Cingulum_Mid_R (290), Cingulum_Mid_L (271), Insula_L (241), Cingulum_Mid_R (236)	
Cerebellum	Cerebelum_6_R (538), Cerebelum_6_L (268), Cerebelum_Crus1_L (266), Cerebelum_Crus1_R (132)	

### Resting State Network Analysis for Aging and the Associated Factors

**FIGURE 4:**
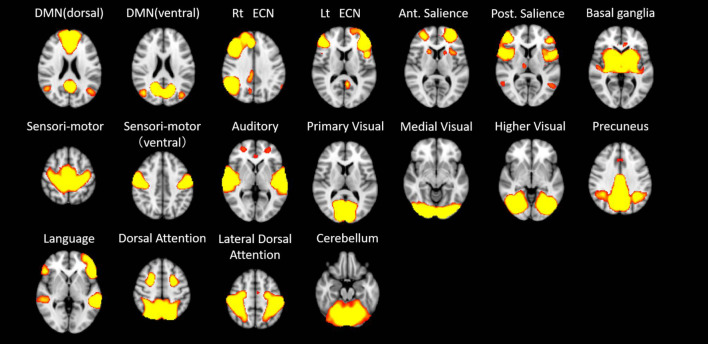
The 18 resting networks extracted at the first step of the dual regression analysis. DMN – default mode network; ECN – executive control network; Rt – right; Ant – anterior; Post – posterior.

**FIGURE 5:**
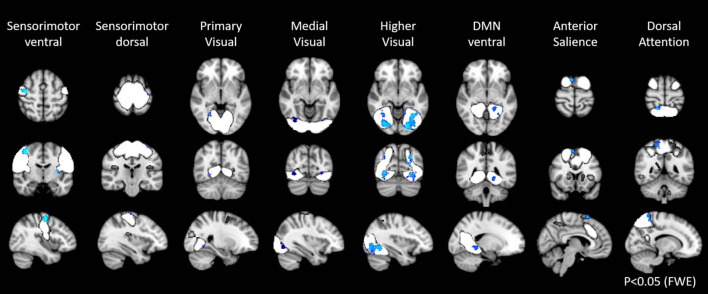
Resting state networks, shown in white, with within-network functional connectivity values that negatively correlated with age. The clusters shown in blue represented the areas with connectivity values showing significant negative correlation with age (FWE *p* < 0.05).

In the first step of the dual regression analysis, 18 resting networks were extracted (Figure 4). Those networks included the ventral and dorsal DMN, the right and left ECN, the anterior and posterior SN, the precuneus network, the dorsal attention network (DAN), lateral DAN, the dorsal and ventral SMN, the basal ganglia network (BGN), the language network (LN), the auditory network, the primary, medial, and higher VN, and the cerebellar network. Out of the 18 networks, eight networks exhibited within-network functional connectivity that was negatively correlated with age (*p* < 0.05, FWE), including the primary, medial, and higher VN, dorsal and medial SMN, DAN, anterior SN, and ventral DMN. The negatively correlated regions in each network were shown in Figure 5, and the anatomical location and voxel counts of those regions were summarized in Table 6. On the other hand, 10 networks did not show significant correlation with age. These networks included the left / right ECN, dorsal DMN, posterior SN, LN, lateral DAN, precuneus, cerebellum, auditory, and BGN.

**TABLE 6:** Anatomical regions decreasing functional connectivity with age in the canonical RSNs.

Primary visual network	Peak MNI coordinate	Peak anatomical region	*P*-value at peak region
	‘26 −54 −6	Lingual_R	*P* = 0.0338
	structures (voxel count):		
	23 Lingual_R (23), Fusiform_R (5)		
Sensorimotor network (dorsal)	Peak MNI coordinate	Peak anatomical region	*P*-value at peak region
	‘−36 −24 68	Precentral_L	*P* = 0.0392
	structures (voxel count):		
	Precentral_L (15)		
Higher visual network (1)	Peak MNI coordinate	Peak anatomical region	*P*-value at peak region
	‘−22 −88 −14	Lingual_L	*P* = 0.0026
	structures (voxel count):		
	Fusiform_L (264), Occipital_Inf_L (241), Lingual_L (190), Occipital_Mid_L (96), Cerebellum_6_L (43), Temporal_Inf_L (8), Temporal_Mid_L (2), Cerebellum_Crus1_L (1)		
Higher visual network (2)	Peak MNI coordinate	Peak anatomical region	*P*-value at peak region
	‘48 −84 8	Occipital_Mid_R	*P* = 0.0016
	structures (voxel count):		
	Fusiform_R (457), Occipital_Mid_R (132), Occipital_Inf_R (124), Lingual_R (90), Cerebellum_6_R (60), Temporal_Mid_R (20), Temporal_Inf_R (2)		
Higher visual network (3)	Peak MNI coordinate	Peak anatomical region	*P*-value at peak region
	‘−14 −52 −8	Lingual_L	*P* = 0.021
	structures (voxel count):		
	Lingual_L (30), Cerebellum_4_5_L (3)		
Higher visual network (4)	Peak MNI coordinate	Peak anatomical region	*P*-value at peak region
	‘−28 −68 34	Occipital_Mid_L	*P* = 0.0066
	structures (voxel count):		
	Occipital_Mid_L (232), Occipital_Sup_L (84), Cuneus_L (41)		
Higher visual network (5)	Peak MNI coordinate	Peak anatomical region	*P*-value at peak region
	‘34 −76 36	Occipital_Mid_R	*P* = 0.0228
	structures (voxel count):		
	Occipital_Mid_R (70), Occipital_Sup_R (11)		
Dorsal attention network	Peak MNI coordinate	Peak anatomical region	*P*-value at peak region
	‘20 −50 66	Parietal_Sup_R	*P* = 0.0162
	structures (voxel count):		
	Parietal_Sup_R (75), Precuneus_R (34), Postcentral_R (21)		
Anterior salience network	Peak MNI coordinate	Peak anatomical region	*P*-value at peak region
	‘6 16 66	Supp_Motor_Area_R	*P* = 0.0162
	structures (voxel count):		
	Supp_Motor_Area_R (57)		
Medial visual network (1)	Peak MNI coordinate	Peak anatomical region	*P*-value at peak region
	‘34 −76 −6	Occipital_Inf_R	*P* = 0.03
	structures (voxel count):		
	Occipital_Inf_R (29), Middle Occipital Gyrus, Occipital_Mid_R (11), Fusiform_R (6)		
Medial visual network (2)	Peak MNI coordinate	Peak anatomical region	*P*-value at peak region
	‘−26 −76 −4	Occipital_Inf_L	*P* = 0.0118
	structures (voxel count):		
	Occipital_Inf_L (12), Occipital_Mid_L (7), Lingual_L (3), Fusiform_L (2)		
Ventral default mode network (1)	Peak MNI coordinate	Peak anatomical region	*P*-value at peak region
	‘−34 −34 −8	Hippocampus_L	*P* = 0.0208
	structures (voxel count):		
	Hippocampus_L (19)		
Ventral default mode network (2)	Peak MNI coordinate	Peak anatomical region	*P*-value at peak region
	‘−18 −42 −6	Lingual_L	*P* = 0.0272
	structures (voxel count):		
	Lingual_L (93), ParaHippocampal_L (19), Precuneus_L (8), Fusiform_L (1)		
Ventral sensorimotor network (1)	Peak MNI coordinate	Peak anatomical region	*P*-value at peak region
	‘−32 −8 6	Insula_L	*p* = 0.013
	structures (voxel count):		
	Insula_L (12)		
Ventral sensorimotor network (2)	Peak MNI coordinate	Peak anatomical region	*P*-value at peak region
	‘ 62 2 34	Postcentral_R	*P* = 0.036
	structures (voxel count):		
	Postcentral_R (14), Precentral_R (10)		
Ventral sensorimotor network (3)	Peak MNI coordinate	Peak anatomical region	*P*-value at peak region
	‘ 44 −8 55	Precentral_R	*P* = 0.001
	structures (voxel count):		
	Precentral_R (161), Frontal_Mid_2_R (66), Frontal_Sup_2_R (11)		

**FIGURE 6:**
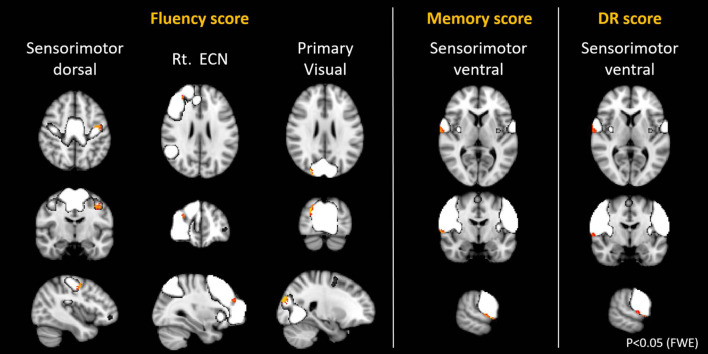
Resting state networks, shown in white, with within-network functional connectivity values that positively correlated with the score of fluency, memory, and delayed recall (DR). The clusters shown in yellow-red represented the areas with connectivity values showing significant positive correlation with the different scores (FWE *p* < 0.05).

With regards to the relationship with cognitive function, the score of the domain of memory and the DR was found to be positively correlated with the SMN (*p* < 0.05, FWE). The regions with positive correlation in the SMN were almost the same in the memory and the DR (Figure 6, Table 7). Furthermore, the score of the fluency was found to be positively correlated with 4 networks, the right ECN, the primary visual, and the dorsal SMN (Figure 6, Table 7). Longer years of education was weakly associated with higher connectivity in the primary visual network, the precuneus, the DAN, and the ventral DMN, and with lower connectivity in the cerebellar network (*p* < 0.05, FWE).

**TABLE 7:** Anatomical regions increasing functional connectivity with cognitive score in the canonical RSNs.

Anatomical regions increasing functional connectivity with Fluency score			
Rt.executive control network	Peak MNI coordinate	Peak anatomical region	*P*-value at peak region
	32 44 26	Frontal_Mid_2_R	*P* = 0.0274
	Structures (voxel count):		
	Frontal_Mid_2_R (18), Frontal_Sup_2_R(4)		
Primary visual network	Peak MNI coordinate	Peak anatomical region	*P*-value at peak region
	26 −90 22	Occipital_Sup_R	*P* = 0.0044
	structures (voxel count):		
	Occipital_Sup_R(60), Occipital_Mid_R(8)		
Dorsal sensorimotor network (1)	Peak MNI coordinate	Peak anatomical region	*P*-value at peak region
	‘−30 −18 40	Precentral_L	*P* = 0.0306
	Structures (voxel count):		
	Precentral_L(2)		
Dorsal sensorimotor network (2)	Peak MNI coordinate	Peak anatomical region	*P*-value at peak region
	‘−40 −8 48	Precentral_L	*P* = 0.0156
	Structures (voxel count):		
	Precentral_L(53), Postcentral_L(31)		

**Anatomical regions increasing functional connectivity with Memory score**			

Ventral sensorimotor network	Peak MNI coordinate	Peak anatomical region	*P*-value at peak region
	‘58 6 −2	Temporal_Pole_Sup_R	*P* = 0.0108
	Structures (voxel count):		
	Temporal_Sup_R(32), Temporal_Pole_Sup_R(30), Rolandic_Oper_R(4)		

**Anatomical regions increasing functional connectivity with DR score**			

Ventral sensorimotor network (1)	Peak MNI coordinate	Peak anatomical region	*P-*value at peak region
	‘60 2 0	Temporal_Pole_Sup_R	*P* = 0.022
	Structures (voxel count):		
	Temporal_Pole_Sup_R(18), Temporal_Sup_R(3)		
Ventral sensorimotor network (2)	Peak MNI coordinate	Peak anatomical region	*P*-value at peak region
	‘64 −8 6	Temporal_Sup_R	*P* = 0.0332
	Structures (voxel count):		
	Temporal_Sup_R(23), Heschl_R(5), Rolandic_Oper_R(4)		

## Discussion

In this study, we evaluated the relationship between aging and cognitive function in a total of 120 healthy subjects consisting of a balanced number of participants within age-groups of 20s, 30s, 40s, 50s, and 70s, who maintained relatively good cognition. Our results were as follows: (1) Among the sub-scores of domains in the cognitive test, the DR, memory, attention/orientation, and visuospatial scores were significantly correlated with age. (2) The score of the DR demonstrated the highest negative correlation with age in this healthy cohort. In the regression analysis, the language and fluency scores did not show significance, whereas other domains (attention/orientation, memory, and visuospatial), and the DR score showed significant relationship with age. A quadratic approximation of the attention/orientation, memory, and the DR scores as functions of age showed relatively delayed decline compared to the total GMV loss. (3) In VBM analysis, widespread brain regions demonstrated negative correlation with age. However, no regions have GM values that correlated with the scores of all domains in the cognitive test when age was included as a covariate. (4) In the analysis for RSNs, although several networks demonstrated a decrease of within-network functional connectivity with age, such a decrease was not observed in 10 networks including the left / right ECN, dorsal DMN, posterior SN, LN, lateral DAN, precuneus, cerebellum, auditory, and BGN. (5) The SMN was positively correlated with the scores of the domain of memory and fluency, and the DR score.

### Morphological Analysis for Aging and Cognition

We selected subjects whose ACE-R score was above the cutoff and was considered normal in cognition. Even in such subjects, ACE-R showed variances in some domains and sub-scores with the DR being the most sensitive sub-score for aging. This finding has a clinical importance to interpret the results of ACE-R. DR purely evaluates short-term memory, not tightly associated with one’s experience, and reflects fluid intelligence. Among the five domains of this cognitive screening test, memory, attention/orientation, and visuospatial ability significantly demonstrated negative correlation with age. On the other hand, language and fluency were not significantly correlated with age. These two domains may not be strongly affected by aging in healthy cohort with a relatively maintained cognition, because language ability is more related to one’s experiences, knowledge, and vocabulary, that is, crystalized intelligence, and fluency also requires such functionality. These findings support the idea that crystalized intelligence is more maintained than fluid intelligence in healthy aging (Baltes et al., 1999).

In VBM analysis, our results showed that the GMV widely declined with age, even starting from the early 20s. This result is consistent with many previous studies (Good et al., 2001; Giorgio et al., 2010; Taki et al., 2011). Regarding the location of regions showing negative correlation with age, the areas around the central sulcus and the intraparietal sulcus were commonly reported in several literatures (Good et al., 2001; Giorgio et al., 2010; Taki et al., 2011), but as to deep brain structures such as the hippocampus, the results differed (Good et al., 2001; Giorgio et al., 2010; Taki et al., 2011; Squarzoni et al., 2018). In our study, we found a significantly lower GMV in bilateral regions around the central sulcus and the intraparietal sulcus, and bilateral medial temporal areas including the hippocampus in older adults. In Alzheimer’s disease, atrophic changes of GMV have been observed in the medial temporal lobe and the temporo-parietal junction. These changes were also frequently observed even in the stage of mild cognitive impairment (MCI) (Baron et al., 2001; Risacher et al., 2009). We adopted 83 as the cutoff of ACE-R in this study (Mathuranath et al., 2000; Yoshida et al., 2012), and our cohort included four individuals whose total score was between 83 and 89. These individuals may potentially be at the prodromal stage of dementia, that is, MCI, and could have influenced our results. The WMV was known to demonstrate a U-shaped change with age (Bagarinao et al., 2018), and therefore, we could not find a linear correlation in our analysis. In VBM, we did not find regions with GMV that correlated with the scores of cognitive domain in ACE-R when age was included as a covariate. This result reflects difficulty to evaluate significant relationship between cognition and morphological changes when simultaneously accounting for the influence of age. In the analysis without age as a covariate, the DR score positively correlated with the GMV of a relatively wider brain region that included bilateral frontal cortices, bilateral temporal cortices, bilateral insular cortices, and bilateral cingulate cortices. We assumed that the function of the DR may require activities in a variety of regions including the hippocampus and the nearby medial temporal structures. However, such a topographic characteristic was not observed in our results. These results should be interpreted with care considering the dependence of both DR and GMV with age. In other words, the affected regions may be “related to the decline of the DR score due to aging” and not to the DR function itself. A study by Takeuchi et al., which examined this relationship using a large sample and age-matched healthy young adults with mean age of 20.8 years and SD of 0.8, reported that there was no strong correlations between regional GMV and specific cognitive domains. Diverse cognitive functions may be weakly associated with regional GMV in widespread brain areas, and may be difficult to detect this association in this analysis.

Regarding the relationship between the morphological changes of the brain and cognition with age, Schnack et al. (2015) reported about the relationship between the intelligence quotient (IQ) and the thickness of the cortex over age. Higher IQ was associated with larger and thicker surface area until around the age of 20, but this relationship weakened from the age of 40 to 50. They also mentioned that individuals maintaining high IQ may form highly efficient formation of brain networks (Schnack et al., 2015). Although they utilized the IQ, which has four domains including the language, working memory, visuospatial, and performance speed, the results was similar to ours. Our results also demonstrated that the GMV decreased with age from a relatively early stage (20’s), whereas the domain of memory and attention, as well as the DR score was maintained to some extent until the late 50s. In healthy aging, a decrease in the GMV and a decrease in cognition showed such temporal dissociation and never showed parallel relationship. The absence of this relationship could not be simply explained by morphological analysis in the brain, and therefore, we supposed that the network analysis was necessary.

More broadly, existing studies have shown that GM continuously declined with age. Thus, it is indeed intriguing that cognitive scores have inverse U-shaped behavior as a function of age, while GMV decreased linearly. Although speculative, this may point to some possible reserve mechanisms at work, where reserve capabilities are accumulated during childhood and young adulthood. The concept of brain or cognitive reserve (Stern, 2002; Satz et al., 2011; Barulli and Stern, 2013) hypothesized the accumulation of neural resources over the years that could lessen the effects of neural decline associated with aging or age-related diseases (Cabeza et al., 2018). Factors such as longer education, greater physical activity, and involvement in demanding leisure activities, among others, affect reserve capacity (Cabeza et al., 2018). This possibly drive the relative preservation in cognitive scores before it peaks and started to decline. Since reserve can also manifest in terms of efficient use of neural resources (Solé-Padullés et al., 2009), large-scale brain networks may also have important roles to play in the maintenance of cognitive functions during aging. To fully understand the association among brain structure, network, and cognition in the aging brain, more studies are needed.

### Network Analysis for Aging and Cognition

**FIGURE 7:**
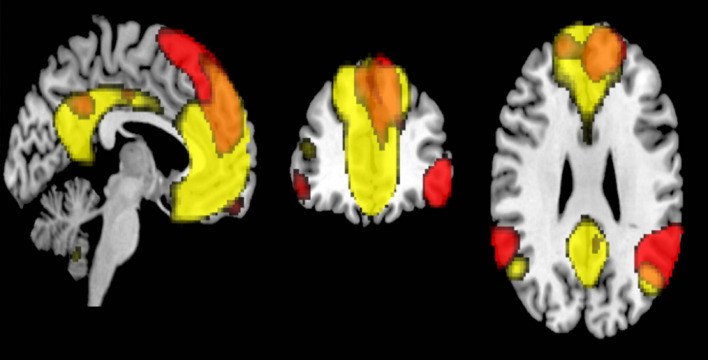
The overlapped regions between the dorsal default mode network (DMN) and the language network (FWE *p* < 0.05).

Previous studies have reported that the connectivity within networks, such as DMN, decreased with age (Damoiseaux et al., 2008; Koch et al., 2010; Jones et al., 2011). Our results also demonstrated similar within-network connectivity decreases in 8 out of 18 RSNs. Specifically, the ventral DMN showed significant decrease in functional connectivity, but not the dorsal DMN. Similar results have been previously reported (Campbell et al., 2013; Huang et al., 2015; Bagarinao et al., 2019). The functional difference between the two is currently not well understood. The ventral DMN is more associated with memory, a hippocampus – dependent function (Damoiseaux et al., 2012), and this network may weaken over age as memory declined. Campbell et al. (2013) examined age-related differences in the intrinsic functional connectivity in subsystems of the DMN. Their findings showed that the subsystem involving dorsal posterior cingulate cortex (PCC) to the fronto-parietal regions was relatively maintained in the elderly, whereas that involving the ventral PCC declined in functional connectivity. The dorsal PCC is a core region in the dorsal DMN, and this could be a reason for the observed discrepancy between ventral DMN and dorsal DMN in our study. With regards to the LN, which also showed no association between connectivity and age, we found regional similarity of its connectivity to that of the dorsal DMN. Both networks shared common regions in the dorsal PCC and dorsomedial prefrontal cortex (Figure 7). The relative maintenance of the dorsal PCC’s connectivity may also explain the relative preservation of LN. In addition, the LN is associated with language ability, an important part of crystalized intelligence. Therefore, this result may be a reflection of the relative maintenance of crystalized intelligence over age. In the absence of supporting literature, more studies examining the association between LN and the network associated with crystallized intelligence are needed. Among the other core cognitive networks, the right / left ECN, lateral dorsal attention, precuneus, and posterior SN have within-network functional connectivity values that did not significantly correlated with age. Previous studies have reported that these networks have decreased within-network functional connectivity (Onoda et al., 2012; Ferreira and Busatto, 2013). However, relative sparing of the functional connectivity in the prefrontal and parietal cortex over age has also been reported (Rajah and D’Esposito, 2005; Reuter-Lorenz and Cappell, 2008; Grady, 2012). These regions are well known to be important for cognitive control, which is a function for the effortful use of cognitive resources to guide, organize, or monitor behavior (Grady, 2012). The networks which were not correlated with age in our study, such as dorsal DMN, the LN, the right / left ECN, lateral dorsal attention, and the posterior salience, included these regions. Some reports suggested that these networks were important for cognitive reserve (Onoda et al., 2012; Chand et al., 2017). Taken together, our results suggest that networks involved with cognitive control were not significantly associated with age. This may reflect the characteristics of our cohort, who had relatively maintained cognition.

Networks associated with primary processing, including ventral and dorsal SMNs and primary VN, demonstrated decrease in functional connectivity with age consistent with previous reports using resting state fMRI and / or task fMRI (Cliff et al., 2013; Roski et al., 2013; Huang et al., 2015; Bagarinao et al., 2019). These results are reasonable considering the vulnerability of the GMV in these areas to aging, physical deterioration, and less external stimulation in the elderly. However, other studies have also demonstrated that the functional connectivity in the primary processing networks is unchanged in advancing age (Geerligs et al., 2015). Therefore, this finding remained inconclusive. The networks related to high-order visual processing also showed negative correlation with age in our study, consistent with previous reports (Yan et al., 2011; Bagarinao et al., 2019).

In terms of the relationship between cognitive functions and functional connectivity within networks, our results demonstrated that the memory score, the DR score, and the fluency score were positively correlated with the SMN. A close relationship between motor function and cognitive function has been reported in behavioral experiments and epidemiological surveys (Clarkson-Smith and Hartley, 1989; Weuve et al., 2004). Recently, a study has reported that physical exercise improved gait speed, and cognitive performance, through the increasing involvement of motor-related networks (Ji et al., 2018). Voss et al. also reported that cardiorespiratory fitness moderated the adverse effects of aging on cognitively and clinically relevant functional brain networks (Voss et al., 2016). Another study also reported that increased functional connectivity of posterior cingulate gyrus / precuneus in individuals with MCI after excise training possibly increase cognitive reserve (Chirles et al., 2017). The neural basis of exercise as an intervention for the maintenance of cognition is being gradually elucidated by recent network analyses, and this may lead to the development of an effective modality about intervention by exercise to prevent cognitive impairment (Huang et al., 2016). The fluency score also showed positive correlation with the right ECN, and weak correlation with the primary VN. Working memory is related to word phonological fluency, and knowledge and vocabulary are related to word categorization fluency (Ruff et al., 1997; Rende et al., 2002; Stolwyk et al., 2015). This association may reflect the relationship between the score of fluency and the ECN. Other cognitive function scores did not show any significant association with the connectivity of any network.

In terms of education history, longer schooling was associated with higher connectivity in the primary VN, the precuneus network, the DAN, and the ventral DMN. The education history was reported to have a correlation with cognitive reserve. In a study with a 4-year follow up, the group with short education history had a 2.2 times higher risk of developing dementia (Stern et al., 1994; Stern, 2009). A more recent study has shown that the risk is 1.5 times higher (Livingston et al., 2017). Given this, long education history plays an important role to keep cognition within normal range, and the neural basis for this may be related to cognition-related networks such as the DAN and the ventral DMN. These networks may have an important role for cognitive reserve. Although the long history of education negatively correlated to the cerebellar network, there has been no report regarding this finding. Recently, there are some reports about detailed analysis for the RSNs in the cerebellum (Dobromyslin et al., 2012; Kawabata et al., 2020). Further study is warranted.

### Limitations

Finally, we enumerated our study’s limitations. First, in the VBM analysis for the DR score, the influence of age could not be completely separated. To identify specific regions related to the DR score using VBM, it is necessary to match the age of all participants and examine individual differences in the DR score. Second, ACE-R is typically used for healthy screening, and has a ceiling effect. Under this limitation, we cannot fully discount its contribution in the observed inverse U – shape behavior in some cognitive domains as functions of age. However, such an inverse U-shape curve is not uncommon in aging studies and has been reported for some cognitive scores (Douaud et al., 2014). Moreover, the sensitivity of the sub-score of ACE-R is not well understood. Therefore, the use of specific cognitive batteries is necessary for a more detailed cognitive evaluation. Third, we just examined the strength of the connectivity within networks. Analysis of the interaction among networks is necessary to fully understand how brain networks contribute to preserve cognition from the GMV loss. There are two important hypotheses, the differentiation (Park et al., 2004; Voss et al., 2008; Goh, 2011) and compensation (Grady et al., 1994; Cabeza, 2002; Davis et al., 2008). To evaluate these mechanisms, the between-network analysis will be performed in a future study. Fourth, this study used cross-sectional data collected by each age-group, not a longitudinal observation of individuals. Finally, the effect of head motion during rsfMRI scanning cannot be completely ruled out especially in aging studies (Kato et al., 2020).

## Conclusion

In our study using a well-balanced healthy cohort in terms of the number of participants and age, we found mixed aging characteristics of brain networks. Among the sub-scores of the cognitive screening test, the DR, memory, attention/orientation, and visuospatial scores were significantly correlated with age, but not language and fluency. Furthermore, the cognitive domains that correlated with age, even the highly correlated sub-scores such as the DR score, showed delayed decline compared to the loss of total GMV. In RSN analysis, the ventral DMN, some networks involving primary processing (the primary VN, the dorsal and ventral SMN), and network related to visual function have within-network connectivity values that negatively correlated with age. On the other hand, some RSNs including the left / right ECN, dorsal DMN, posterior SN, LN, and lateral DAN, have within-network connectivity values that were maintained with age in this cohort. This may reflect a relative preservation in cognitive control function and crystalized intelligence in our cohort. Furthermore, the score of memory, fluency, and the DR was correlated with the sensorimotor network, which supported the importance of the exercise for maintenance of cognition.

## Data Availability Statement

The datasets presented in this article are not readily available because of privacy and ethical restrictions. Requests to access the datasets should be directed to SMa, smaesawa@med.nagoya-u.ac.jp.

## Ethics Statement

The studies involving human participants were reviewed and approved by The Ethics Committee of Nagoya University Graduate School of Medicine (approval number 2014-0068). The patients/participants provided their written informed consent to participate in this study.

## Author Contributions

SMa, SMi, EB, HW, MH, HI, NO, MK, RS, and GS contributed to conception and design of the study. SMa, SMi, DM, DN, KH, KK, RO, AO, MH, and HI were involved in data acquisition, data organization, and data curation. SMa, SMi, EB, HW, and KK contributed to the methodology, analysis, interpretation of the data, and wrote the draft of the manuscript. All authors reviewed and approved the final version of the manuscript.

## Conflict of Interest

The handling editor declared a past co-authorship with one of the author GS. The remaining authors declare that the research was conducted in the absence of any commercial or financial relationships that could be construed as a potential conflict of interest.

## Publisher’s Note

All claims expressed in this article are solely those of the authors and do not necessarily represent those of their affiliated organizations, or those of the publisher, the editors and the reviewers. Any product that may be evaluated in this article, or claim that may be made by its manufacturer, is not guaranteed or endorsed by the publisher.

## Footnotes

1
https://www.alivelearn.net/xjview/

2
http://findlab.stanford.edu/functional_ROIs.html
